# The Effect of the Addition of Polypropylene Fibers to Primer on the Pull-Off Strength of Epoxy Resin Coatings

**DOI:** 10.3390/ma13204674

**Published:** 2020-10-20

**Authors:** Łukasz Kampa, Agnieszka Chowaniec, Aleksandra Królicka, Łukasz Sadowski

**Affiliations:** 1 Department of Building Engineering, Wroclaw University of Science and Technology, Wybrzeże Wyspiańskiego 27, 50-370 Wroclaw, Poland; lukasz.kampa@pwr.edu.pl (Ł.K.); agnieszka.chowaniec@pwr.edu.pl (A.C.); 2 Department of Metal Forming, Welding and Metrology, Wroclaw University of Science and Technology, Wybrzeże Wyspiańskiego 27, 50-370 Wroclaw, Poland; aleksandra.krolicka@pwr.edu.pl

**Keywords:** coating, epoxy resin, pull-off strength, polypropylene fibers

## Abstract

This article describes the effect of adding polypropylene fibers to primer on the pull-off strength of epoxy resin coatings. Investigated primers were laid on substrates made of cement mortar and cement slurry. The primer was made of epoxy resin modified with the addition of 0.5%, 1%, 1.5% and 2% of polypropylene fibers. One reference sample was made without the addition of fibers. Then, an epoxy resin coating was applied to each substrate. Four pull-off strength tests were performed for each material configuration. For this purpose, an automatic device for measuring the pull-off strength of the coatings was used. The results were compared with the results obtained for the reference sample. The optimum content of polypropylene fibers was found to be in the range of 0.5–1.0 wt. % of the mass of the resin. One percent of fibers was optimum for the epoxy resin laid on the cement slurry, while 0.5 wt. % of fibers was optimum for the mortar substrate. The addition of a higher amount of polypropylene fibers resulted in a lower pull-off strength of coatings than for the reference sample.

## 1. Introduction

**Figure 1:**
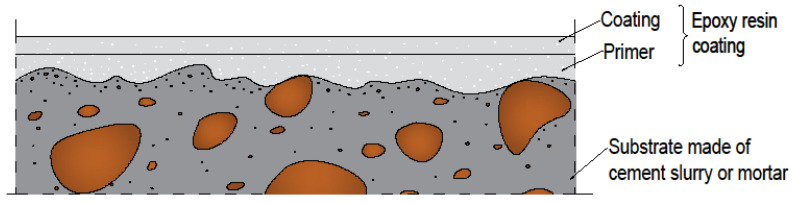
Cross-section of a typical industrial floor made of epoxy resin coating.

Polypropylene fibers and epoxy resins are very popular materials in floor technology [1,2,3,4,5]. However, polypropylene fibers are mainly used in concrete [6]. A typical industrial floor is composed of epoxy resin coating that is placed on the substrate (Figure 1). It is visible from Figure 1 that before the coating can be applied, it is necessary to apply the primer. 

When applying epoxy resin, it is required that the substrate has a minimum compressive strength of 25 MPa and a pull-off strength of no less than 1.5 MPa (there are many modern methods to study this parameter [7]). If the substrate is too weak, it should be strengthened. For this purpose, the surface of the substrate must be mechanically treated. According to [8], grinding is the most effective method of mechanical treatment with regards to the application of epoxy resins. After grinding, the primer is usually applied. It penetrates the substrate well, tightly filling all pores, and thus strengthening the substrate. After hardening, it usually becomes a colorless protective film. Wang et al. [9] and Szymanowski [10] highlighted the strong relation between the protective properties of coatings and overlays and the loss of adhesion as a function of aging processes and durability. Won et al. [11] stated that long-term exposure to water affects the adhesion between the epoxy resin coating and the concrete substrate. Kim et al. [12] studied the early-age tensile bond characteristics of epoxy coatings for underwater applications. Application of the primer is necessary in order to obtain the required value of pull-off strength of the coating (usually a minimum of 1.5 or 2 MPa). However, the required value of pull-off strength is sometimes much higher, and in this situation most primers cannot be applied. 

There have therefore been recent attempts to concentrate on the modifications of epoxy resins using different additives. Many authors are working on the modifications of epoxy resin coatings. Asfhar et al. [13] used various primers and coatings to increase the corrosion resistance of rebars in concrete modified with different additives. They found that polyurethane coating has the highest strength and the lowest corrosion rate, while concrete with polypropylene fibers has the highest compressive strength. De la Varga [14] developed nanosilica coatings to improve the pull-off strength of cementitious materials. Morshed et al. [15] showed that the addition of nanosilica and multi-wall carbon nanotubes to epoxy resin has a positive effect on the bonding between fiber-reinforced polymer composites and concrete. Research [16] investigated the influence of the SiO_2_-graphene hybrid oxide in the resin, and found out that it improved the corrosion resistance of the coating. The adhesion and contact of water with the coating increased. On the other hand, ultrafine fiberglass improves the mechanical and anti-corrosive properties of epoxy coatings [17]. The addition of fiberglass in an amount of 30% made a significant impact on the hardness and adhesion of these coatings when compared to its absence. In some cases, the surface of the substrate was modified with the use of texturing to increase the pull-off strength of coatings [18]. The results of study [18] show that the most effective texturing method involves imprinting crosses in the shape of a “+”. However, this method requires appropriate care in terms of shape, placement and texture depth. Moreover, the addition of waste glass powder to the resin layer allows the required layer adhesion to be obtained without mechanical treatment of the substrate [19]. A similar effect was achieved by adding carefully selected polymers [20], monomers [21], nanomaterials [22] or carbon nanotubes [23]. In [24], Al-Turaif showed that the addition of nano-TiO_2_ to epoxy resin causes a slight improvement in pull-off strength. Resin coatings with a 3D effect are attracting more and more interest among individual customers. This effect is obtained by embedding properly prepared graphics, decorative pebbles, rocks, etc. in the resin, which do not react with the resin used. Even if the number of modifications of the epoxy resin coating is quite large, there has been no attempt to modify the primer (e.g., using polypropylene fibers). The resin coating, after the resin is combined with a solvent as a result of the Diels-Adler reaction, becomes a layer that protects the concrete substrate against corrosion and increases its durability [25]. It caused a reduction in viscosity, thanks to which the resin can penetrate into the concrete by up to even 1.5 mm. It also improves adhesion and mechanical strength. Lower water absorption and the chloride diffusion coefficient are the result of the modification of the resin coating with graphene oxide [26]. Researchers are also trying to modify the material of the primer. Atta et al. [27] used a modified chemical structure of epoxy cardanol as a green bio-based organic primer for steel coatings. Kim et al. [28] developed a primer and an aminosilane-modified epoxy impregnation for bonding fiber-reinforced polymers to concrete in underwater conditions and achieved a pull-off strength of at least 2.0 MPa. In recent decades, more and more interest has been devoted to testing the pull-off adhesion between epoxy coatings and concrete substrates and pull-off strength of coatings [29,30]. Nowadays, there is a lack of research concerning the modification of a primer for the purpose of increasing the pull-off strength of epoxy resin coatings.

When carrying out the literature research conducted so far, it was noticed that polypropylene fibers are a frequent additive to epoxy resins [31]. In an article [32], Dutra et al. showed that the presence of ethylene–vinyl acetate copolymer modified with mercapto groups in the polypropylene fibers improves its adhesion to the epoxy resin. The results were confirmed by scanning electron microscope (SEM) JEOL JSM-6610A (Tokyo, Japan). A higher impact resistance and glass transition temperature of this composite were also obtained. In [33], Dutra et al. showed that the modification of epoxy resin with polypropylene and carbon fibers improves the impact resistance and increases the glass transition temperature without affecting the thermal stability. Prabhu et al. [34] investigated the thermal degradation of epoxy resin reinforced with polypropylene fibers. These tests showed that the addition of polypropylene fibers increases the thermal stability of the epoxy resin. As in these research, they performed a scanning electron microscope analysis which showed good dispersion of fibers in the matrix. In [35], an epoxy resin with addition of clay and short polypropylene 3mm length fibers were investigated. The addition of untreated polypropylene fibers to the epoxy resin deteriorated the tensile, flexural and impact strength. This decrease in the mechanical properties of the composite was due to the poor compatibility of the polypropylene fibers with other components. By using the compatibilization procedures (silane treatment and maleic anhydride grafting) of polypropylene fibers, a composite with better impact strength was obtained without a significant decrease in tensile and flexural properties. As can be seen from the quoted research results, thanks to the addition of polypropylene fibers to the epoxy resin, it is possible to improve some of its parameters.

Considering the above, this article studies the effect of the addition of polypropylene fibers to primer on the pull-off strength of epoxy resin coatings. The substrates were cement slurry and cement mortar, and in this research, 0.5%, 1%, 1.5% and 2% of polypropylene fibers were used. A reference sample without polypropylene fibers was also made. The obtained results were compared with the reference sample.

## 2. Materials and Methods

### 2.1. Substrates

**Figure 2:**
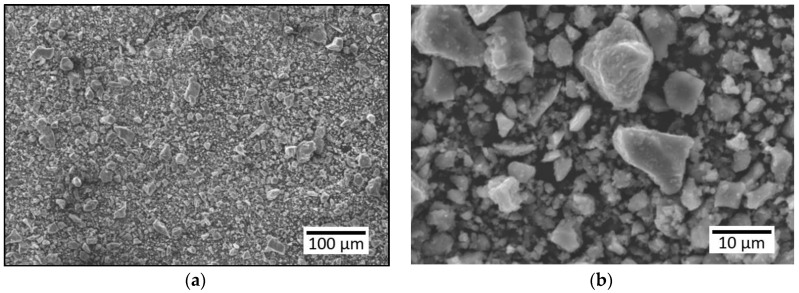

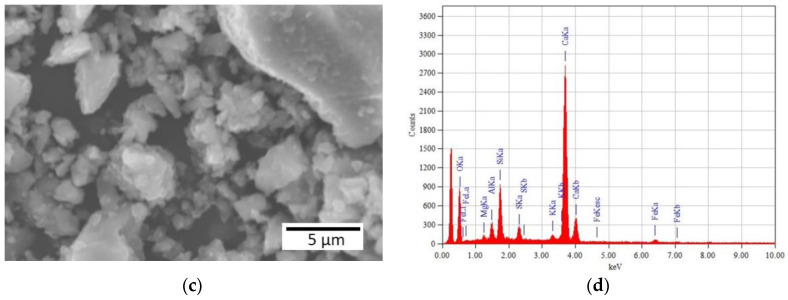
Morphology (**a**,**b**,**c**) and X-ray diffraction spectrum of the cement used to make the substrate (**d**).

Two types of substrates were prepared. One type was made using cement mortar, and the second type with cement slurry. The Portland cement class I 42.5 R was used for both substrates and has the following characteristics: composition: 95–100% Portland clinker, 0–5% secondary components; compressive strength: early ≥ 20 MPa, standard ≥ 42.5 MPa and ≤ 62.5 MPa; setting time (beginning) ≥ 60 min; ignition loss ≤ 5%; expansion ≤ 10 mm; SO_3_ content ≤ 4%; chloride content ≤ 0.1%. Figure 2 shows the morphology and X-ray diffraction spectrum of the cement used to make the substrate.

Based on the chemical analysis, determined using an energy-dispersed x-ray spectrometer (EDS, EDX), it was found that the cement consists of Si (53.0 wt %), Al (16.5 wt %), Ca (12.5 wt %) and Fe (8.0 wt %). The content of other components (Na, Mg, K) does not exceed 4.0 wt % (Figure 2d). Observations of the morphology of the tested cement were performed using scanning electron microscopy and topographic contrast (secondary electrons—SE detector). The results are presented in Figure 2a–c. 

**Figure 3:**
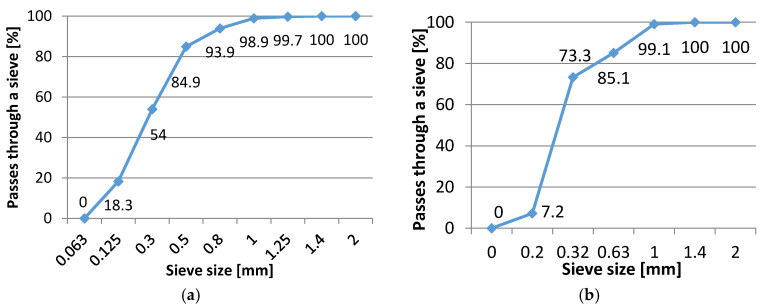
Sieve size of: (**a**) cement used to make the substrate, (**b**) fine aggregate used to make the substrate made of cement mortar.

Preparation of the sample consisted of measuring all the dry ingredients and then mixing them together in a mixer. The mortar consisted of cement, dried quartz sand and water in the proportions 1:3:0.5 (w/c = 0.5), and the slurry was made with the ratio w/c = 0.4. After mixing the dry ingredients, the pre-measured amount of water was added, and then mixed for 90 seconds. After the first mixing, the ingredients that settled on the walls of the mixer were mixed by hand, and then mixed again in the mixer for 90 seconds. After these tests, the mixture was placed in previously prepared wooden molds with dimensions of 150 × 150 × 40mm. The mixture placed in the molds was covered with foil for 24 hours. After this time, the samples were removed from the molds and then left for 28 days to cure. The samples were dried in air at a temperature of 20–23 °C and a humidity of 60–65%. After full curing, the surface of the samples was ground to an even surface. Figure 3 presents the sieve size of cement used to make the substrate and fine aggregate used to make the substrate made of cement mortar.

### 2.2. Polypropylene Fibers

Polypropylene “Fiber MicroArm” fibers were used for the test. Their diameter, declared by the manufacturer, is 0.02 mm ± 10%, tensile strength is 360 ± 7.5% N/mm^2^, linear density is 0.3 ± 0.5 tex, specific gravity is 0.91 kg/m^3^, Young’s Modulus is 3500 MPa and the softening point is 120 °C. The manufacturer declares chemical resistance to acids, bases and solvents.

**Figure 4:**
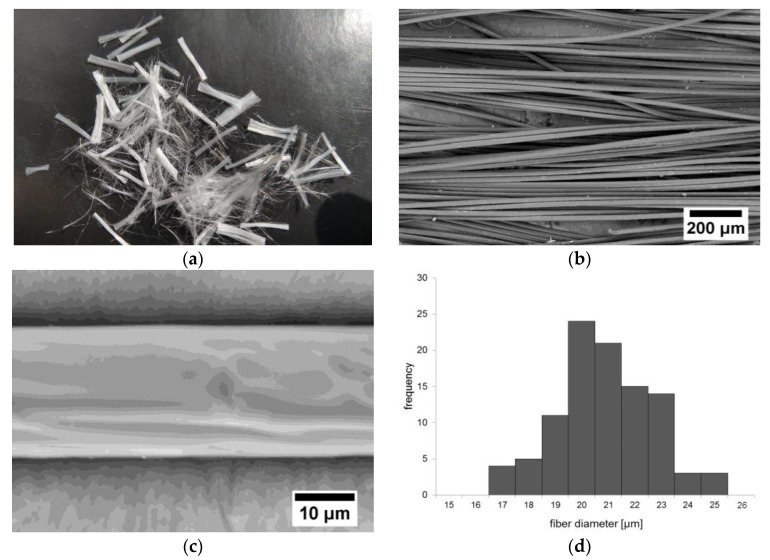

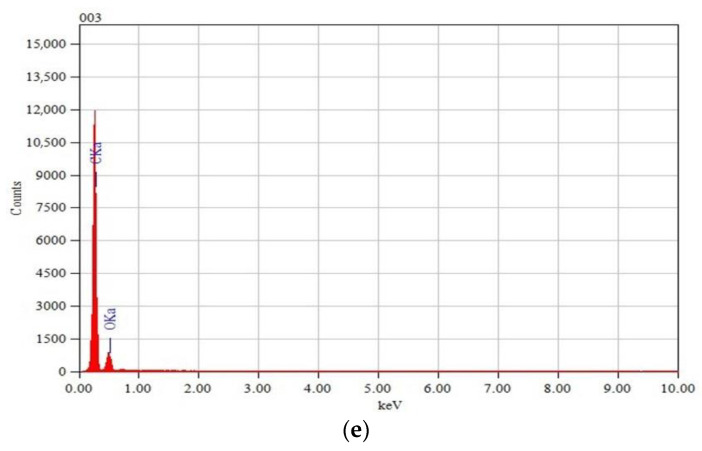
Polypropylene fibers used in the research: (**a**) macrophotograph of the fibers, (**b**) microphotograph of the fibers (using scanning electron microscopy (SEM) and the backscattered electron (BSE) detector), (**c**) surface topography of the fibers (using SEM and the BSE detector), (**d**) histogram of the fibers’ diameters, (**e**) X-ray diffraction spectrum obtained from the area presented in Figure 4c.

The distribution of the probability of the occurrence of a given fiber diameter was determined on a sample of 100 randomly selected fibers. Measurements were made using SEM images and ImageJ software (version 1.51w, National Institutes of Health, Bethesda, MA, USA). The measurement results are presented in Table 1. The histogram of the fibers’ diameter exhibits an approximately normal distribution (Figure 4d). Based on the obtained measurement results, it was found that the diameters of the fibers generally meet the requirements presented by the manufacturer (x—20.4 ± 1.8 μm; median—20.3 ± 1.1 μm).

Polypropylene fibers are delivered in the form of unidirectional bundles (Figure 4a) with lengths in the range of 10–12 mm. The surface of the fibers was characterized by a smooth surface, which is typical for polymer fibers (Figure 4b,c). They were in the form of groups that were difficult to break up. A greater amount of fibers in the resin caused it to become thickened (the resin “surrounded” each fiber). Based on the analysis of the chemical composition determined using an energy-dispersed x-ray spectrometer (EDS, EDX), the presence of carbon and oxygen was found (Figure 4e). It should be noted that the EDS method allows the identification of chemical elements with a mass number higher than carbon. For this reason, it was not possible to quantify the content of oxygen, carbon and hydrogen. The identified presence of carbon and oxygen in the X-ray diffraction spectrum is typical for polymers. Thus, the analysis of the chemical composition confirmed the polymer origin of the tested fibers. 

### 2.3. Epoxy Resin

An epoxy resin primer (Meteor Primer by Si-Tech Sp. z o. o., ul. Dobra 9, 05-306 Jakubów, Poland), characterized by a density of 1–1.2 g/cm^3^, a low viscosity of 400–600 MPa·s, a usability of 20–30 min and full hardening after 24 hours, was used for the test. The top layer was Meteor Stone from the same manufacturer. Its density is 1.15–1.25 g/cm^3^ and its viscosity is 750–1000 MPa·s. It has a shelf life (at 20 °C) of 20–25 min and is fully hardened after 24 h. Both resins are the result of the reaction of bisphenol A with epichlorohydrin (epoxy resin—average molecular weight ≤ 700). The resins have the following parameters: reaction to fire—Bfl-s1, release of corrosive substances—SR, abrasion resistance of ≤ AR1, impact resistance of ≥ IR4 and pull-off strength of ≥ B1.5 (manufacturer data). The first resin layer, which was made using Meteor Primer resin (Si-Tech Sp. z o. o., ul. Dobra 9, 05-306 Jakubów, Poland), was modified in the study. The modification procedure is provided in the next section.

### 2.4. Preparation of Samples

**Figure 5:**
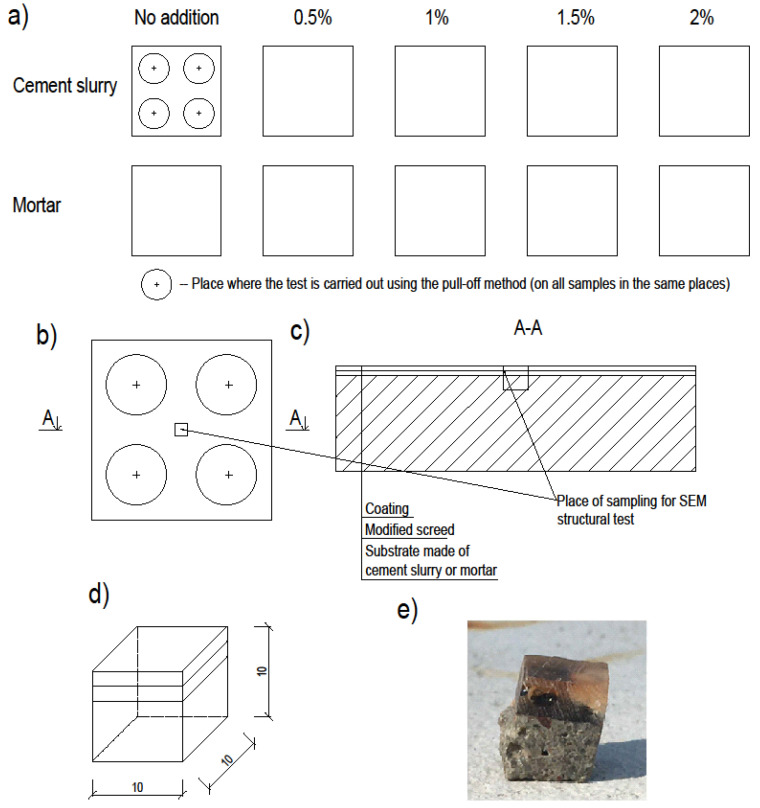
Samples prepared for testing: (**a**) division of samples into test fields, (**b**) a single sample with the place of sample collection for the SEM structural test, (**c**) cross-section through the sample, (**d**) a cut cube with dimensions of 10 × 10 × 10 mm subjected to the SEM structural test, (**e**) photo of the cut sample for SEM testing.

The previously made substrates were taped with gray repair tape. It prevented the resin from pouring out of the sample. The application of resin was preceded by measurement of all the ingredients. The resin consisted of components A and B (100/50 ratio). It was decided to use a fiber addition of 0.5, 1, 1.5 and 2 wt %. All ingredients were weighed in plastic buckets. Fibers were poured into the bucket with measured component A and everything was mixed with a metal rod. After that, the mass clearly thickened (especially with a higher amount of additive). The hardener (component B) was then added and mixed for approximately 2 min. The entire mixture was poured over the substrates while ensuring the even distribution of the fibers. Due to its density, it was quite difficult to obtain a more even resin surface. The final thickness of the layer was about 2 mm. A control test, without the addition of fibers, was also performed. Figure 5 presents samples prepared for testing:

After a few hours, the topcoat of Meteor Stone was applied. In this case, the proportion of components A and B is 100 to 50 by weight. The surface resin was prepared in an amount that was necessary to make a 2 mm thick layer on all 10 samples at the same time. 

The preparations commenced approximately 10 days after the application of the resin. Unfortunately, resin leakage was found in some samples and air bubbles in the resin formed on the surface of four samples. Any unevenness remaining after applying the resin was sanded with a grinder with a diamond disc for grinding concrete. The surface temperature of the sample remained unchanged.

### 2.5. Pull-Off Strength Test

**Figure 6:**
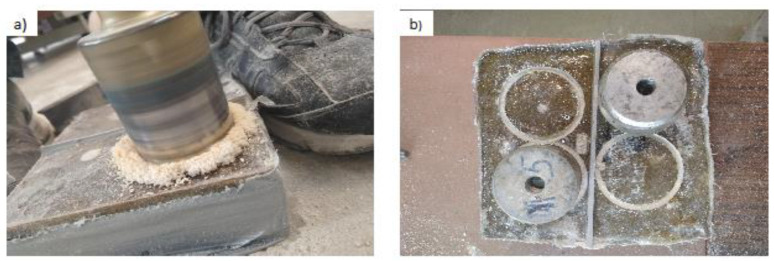
View of: (**a**) drilling holes for discs, (**b**) glued test discs.

**Figure 7:**
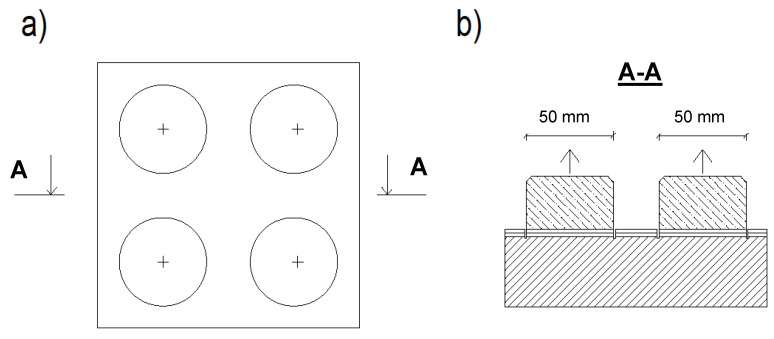
Diagram of the arrangement of the discs on the sample: (**a**) view, (**b**) cross-section.

The sample was then measured in such a way that it was possible to carry out four trials (two per layer). The center of the hole was about 7.5 cm from the edge of the sample. The holes were made with a diamond concrete hole saw with an inside diameter of 5 cm at a depth just below the resin layer. The drilling was conducted with the aid of a device that stabilized the drill in position. During the drilling itself, the speed of the drill was kept low and the hole saw was cooled down with water every few seconds. However, the surface of the sample in the area where the hole saw was spinning had a higher temperature of resin in its vicinity. After drilling all the holes, the surface was cleaned with a soft brush and washed with a cloth soaked in acetone. Figure 6 shows the view of drilling holes for discs and glued test discs, while Figure 7 presents the diagram of the arrangement of the discs on the sample.

The test itself began with the preparation of an adhesive force measuring device, which was connected to the substrate with steel discs, the surface of which was first washed with acetone. After that, they were glued to the sample with the epoxy resin Epidian 5 with the hardener Z1 produced by Ciech Sarzyna (Nowa Sarzyna, Poland) in the proportion of 100 to 12. The test was divided into two rounds. In each of them, two discs were glued on the diagonal of the sample.

The places where the test was carried out had to have a flat surface. Perimeter sites were not selected as they may not be representative of the entire sample. In accordance with standard [36], to characterize the area, at least three breaks had to be made, the distance between each individual one being large enough to position the device. To reduce the risk of damage to the glue and increase its adhesion, the surface was sanded so as not to damage it or change its thickness. After that, the sample and the disc were washed with a solvent that does not endanger the integrity of the coating (acetone). Glue was applied to both surfaces and was carefully spread over the entire surface. The adhesive used did not affect the coating. After applying the disc, the excess glue was carefully removed, taking care not to let air get between the disc and the coating (be careful of twisting). Constant pressure is provided at the beginning of the bonding of the adhesive. After the glue is fully cured, the test can begin. The element connecting the apparatus with the disc should be carefully screwed into it, and the spherical end should be mounted in the jaw of the device. After the device is zeroed and levelled, it must be programmed to complete the test in approximately 100 seconds or less. The result was recorded on the device, along with a graph of the load increment over time.

For the tests, the Proceq dy-216 apparatus was used, which, after entering appropriate data, automatically determined the peel strength and created graphs of strength versus time. The camera load speed was 0.05 MPa/s.

### 2.6. Ultrasonic Testing

**Figure 8:**
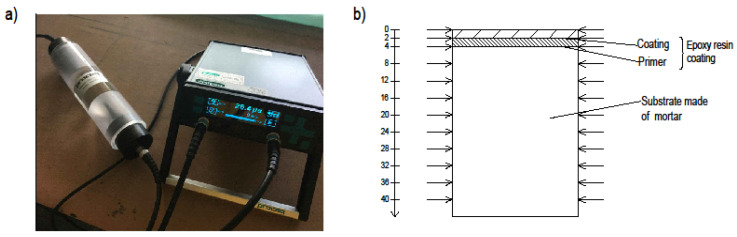
Ultrasonic testing: (**a**) device accuracy test, (**b**) diagram of measuring points on the sample’s cross-section.

A study was also performed to measure the velocity of ultrasounds through the sample, which made it possible to burn off the degree of densification of the substrate and the resin. The faster the ultrasound flowed, the greater was the material filling. The Proceq pundit lab (Proceq, Schwerzenbach, Switzerland) was used in this study. The samples were properly prepared—they were cut into two parts and the opposite sides were grinded so that they were parallel. Figure 8 shows the device accuracy test together with the diagram of measuring points on the sample’s cross-section used in performed ultrasonic testing.

Before starting the measurements, a measuring stand was prepared and the accuracy of the device was tested according to the manufacturer’s recommendations (Figure 8a). Twelve measurements were made on each sample (Figure 8b), with the first (0 mm) being on the coating at the edge of the sample. The next measurement (2 mm) was performed at the interphase between the coating and primer. At a depth of 4 mm, the interphase between the primer and the substrate was tested. The remaining nine measurements (8–40 mm) were made every 4 mm in the cement mortar substrate.

### 2.7. SEM Chemical and Microstructural Analysis

**Figure 9:**
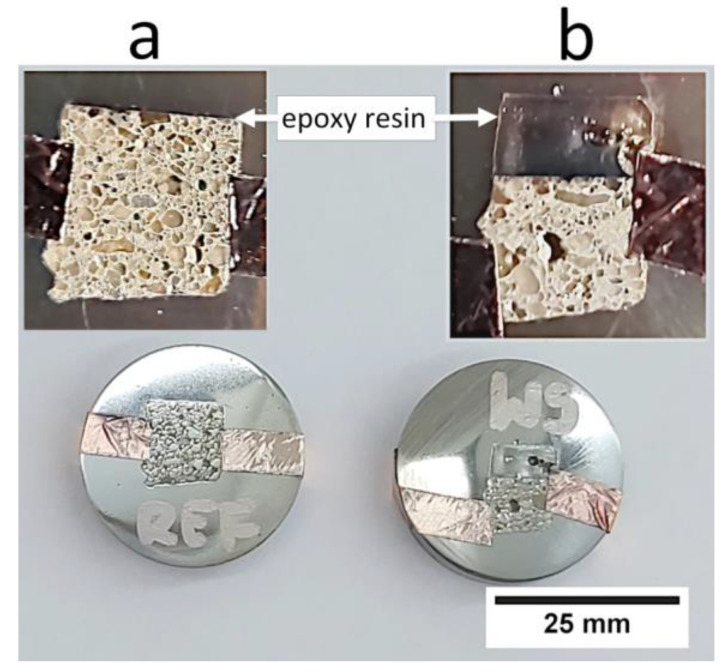
Prepared samples for testing microstructure with a carbon coating: (**a**) reference sample without added fibers, (**b**) sample modified with 0.5 wt % of added fibers.

The SEM JEOL JSM-6610A (Tokyo, Japan), equipped with tungsten filament, was used for the microstructure analysis. The material contrast mode of the back-scattered electrons (BSE) detector, a beam current of 41 nA, an accelerating voltage of 20 kV and a working distance of 10 mm were used for the microstructure observations. The samples for investigations were immersed in a non-conductive epoxy resin and then ground (by sandpaper with a number of 320–4000) and polished (by diamond medium with a size of 1 μm). To obtain electrical conductivity and to avoid electrical charging of the cross-section of the samples, a conductive path made of copper tape was used and the samples were then coated with carbon. A conductive carbon layer with a thickness of 25 nm was applied in a vacuum of 2 × 10^−5^ Pa (2 × 10^−6^ Torr) by thermal sputtering from graphite electrodes. The prepared samples for the microstructure investigations are presented in Figure 9.

The chemical element composition was determined using an energy-dispersed x-ray spectrometer (EDS, EDX) JEOL JED-2300 (Tokyo, Japan). The count rate was set to approximately 5000 cps and the detector dead time did not exceed 15%. Quantitative analyses were performed using JEOL software (JEOL JED-2300 Analysis Station) and the integrated ZAF method (atomic number effect (Z), self-absorption effect (A) and fluorescence effect (F)). Elementary mappings were performed to the visualization of the relative chemical composition distribution of the tested samples. The data acquisition time was about 1 h for one analyzed area. The obtained intensity maps of the elements had a resolution of 512 × 384 points with the size of the observation field being 135 × 100 μm.

## 3. Results

### 3.1. Pull-Off Strength of Coatings

**Figure 10:**
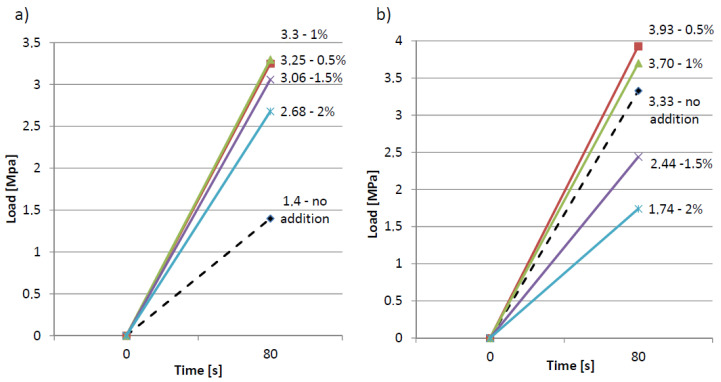
Load rate during the pull-off test of the epoxy resin coating from: (**a**) the cement slurry, (**b**) the mortar.

In five primers, the resin remained wet after about two weeks from being applied. About 80% of the sample surface was detached in one sample. The remainder was breaking off the backing. The strength versus time graphs give us an almost linear function in each case. Figure 10 presents load rate during the pull-off test of the epoxy resin coating from the cement slurry and the mortar.

**Figure 11:**
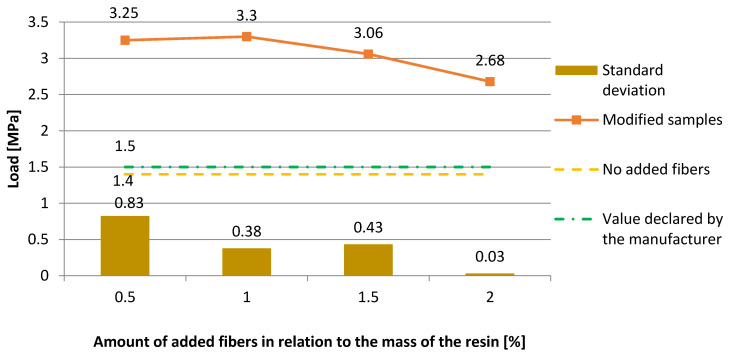
The dependence of the pull-off strength of the epoxy resin coating on the cement slurry substrate with regards to the amount of added fibers.

Figure 11 presents the dependence of the pull-off strength of the epoxy resin coating on the cement slurry substrate with regards to the amount of added fibers. Figure 11 shows that the highest values of pull-off strength equal to 3.3 MPa and 3.25 MPa were obtained with the addition of 1% and 0.5% of fibers, respectively (standard deviations of 0.38 MPa and 0.83 MPa). Above 1% of additive, a downward trend can be seen—the grip is decreasing. The test of the reference sample showed a result of 1.4 MPa, while the manufacturer’s declared pull-off strength was 1.5 MPa. 

**Figure 12:**
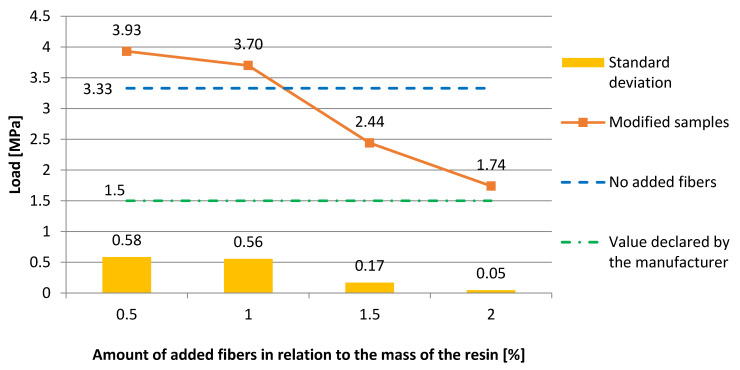
The dependence of the pull-off strength of the epoxy resin coating on the mortar substrate with regards to the amount of added fibers.

Figure 12 shows the dependence of the pull-off strength of the epoxy resin coating on the mortar substrate with regards to the amount of added fibers. From Figure 12, a downward trend can be seen from the lowest additive value. The result of 3.93 MPa (σ = 0.58 MPa) is the best one obtained in the study (0.5% addition). The addition of 1 wt % (3.7 MPa, σ = 0.56 MPa) was also above the value obtained in the reference sample (3.33 MPa). All the tested additives were above the manufacturer’s declared pull-off strength (1.5 MPa). The best result obtained in the entire test may result from both the sufficient liquidity of the resin, which evenly filled the pores on the surface of the mortar, and the fact that the fibers adequately strengthened the proper resin layer.

**Figure 13:**
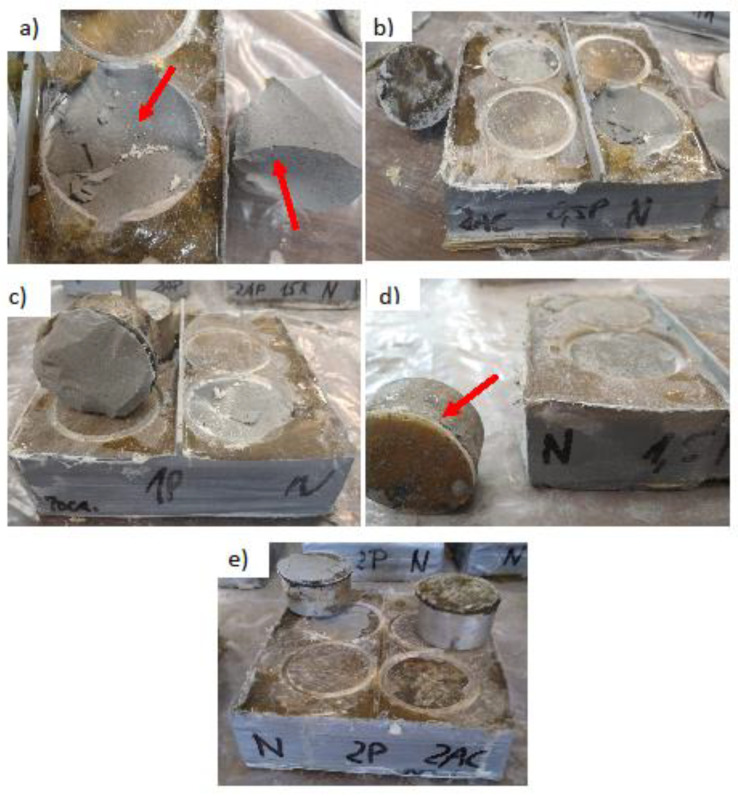
Photos of epoxy resin coating samples after detachment: (**a**) without additive (cohesive failure), (**b**) 0.5 wt % of the additive (adhesive failure), (**c**) 1 wt % (cohesive failure), (**d**) 1.5 wt % (adhesive failure), (**e**) 2 wt % (adhesive failure).

Figure 13 shows selected photos of the characteristic shapes of the detachment surfaces. Out of 20 such places, only in three was the resin layer detached from the substrate (adhesive failure) (Figure 13e). In the rest of the cases the substrate was damaged (cohesive failure). The slurry generally left sharp edges (Figure 13a) and small splinters of grout in the resulting depression. There was no difference between the shape and place of the detachment of the sample and the value of pull-off strength.

**Figure 14:**
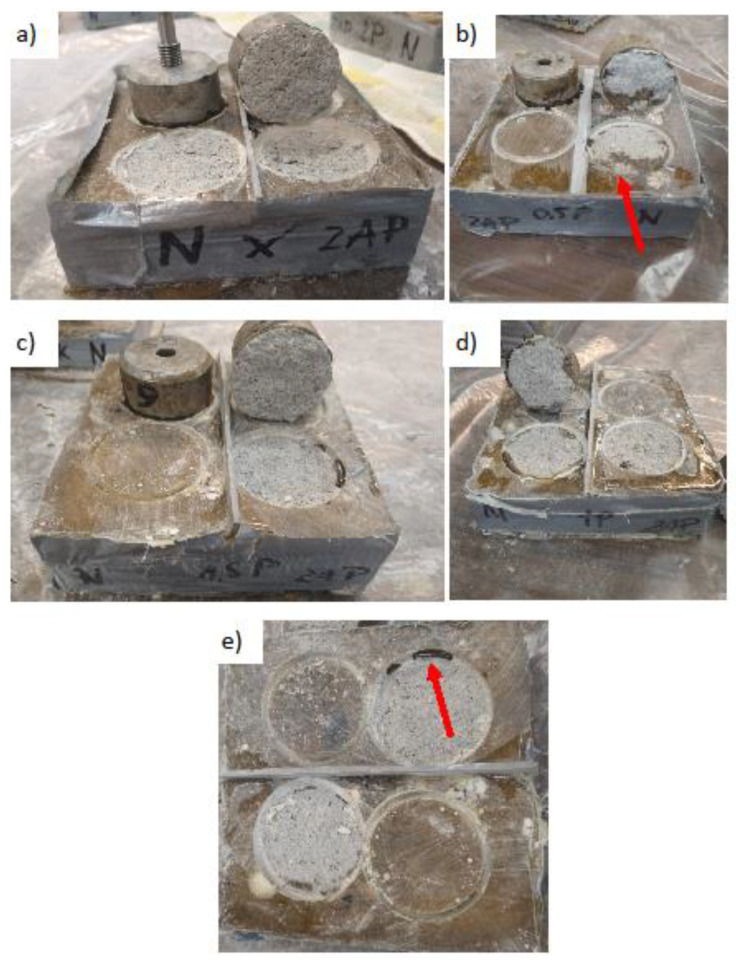
Photos of the mortar samples after detaching: (**a**) without additive (cohesive failure), (**b**) 0.5 wt % (adhesive failure), (**c**) 1 wt % (cohesive failure), (**d**) 1.5 wt % (cohesive failure), (**e**) 2 wt % (cohesive failure).

Figure 14 shows photos of the characteristic surfaces of the mortar after the test. In all the samples, the substrate was damaged at the joining of the aggregate grain with the surrounding matrix. On some of the samples, it had a mixed character (Figure 14b), where the substrate was partially destroyed and the resin layer partially damaged. In several samples, when the steel discs were glued, too much glue was used and it leaked into the groove that was formed during drilling (Figure 14e). However, this did not affect the result of the pull-off strength test.

### 3.2. Ultrasonic Testing

**Figure 15:**
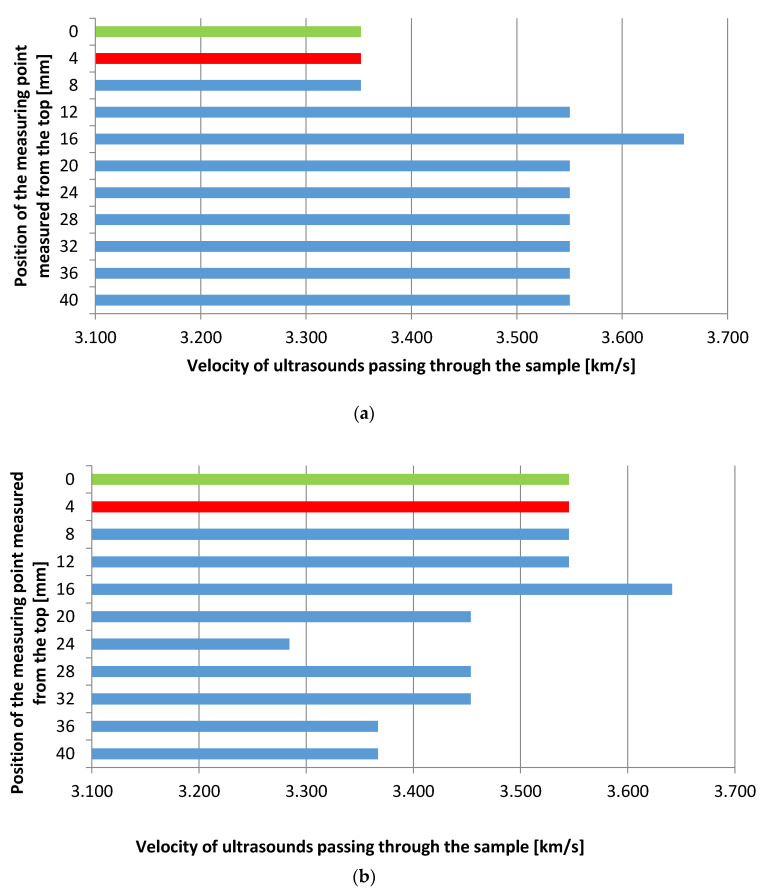
The dependence of the velocity of ultrasounds as a function of the sample’s height (green value—coating, red value—primer, blue value—substrate): (**a**) without the addition of fibers, (**b**) with the addition of 0.5 wt % of polypropylene fibers.

Figure 15 shows the dependence of the velocity of ultrasounds as a function of the sample’s height. The higher the speed, the tighter the sample filling. The most important measurement is the one in the area of applied energy (0–8 mm), because this is where the resin has filled all the pores that formed during the application of the primer (green value—coating, red value—primer, blue value—substrate). It can be concluded from the above figure that the sample without polypropylene fibers filled the pores in the resin area better—the speed turned out to be higher (3.55 mm/μs) than in the case of adding 0.5 wt % of the fiber (3.35 mm/μs). 

### 3.3. SEM Chemical and Microstructural Analysis

**Figure 16:**
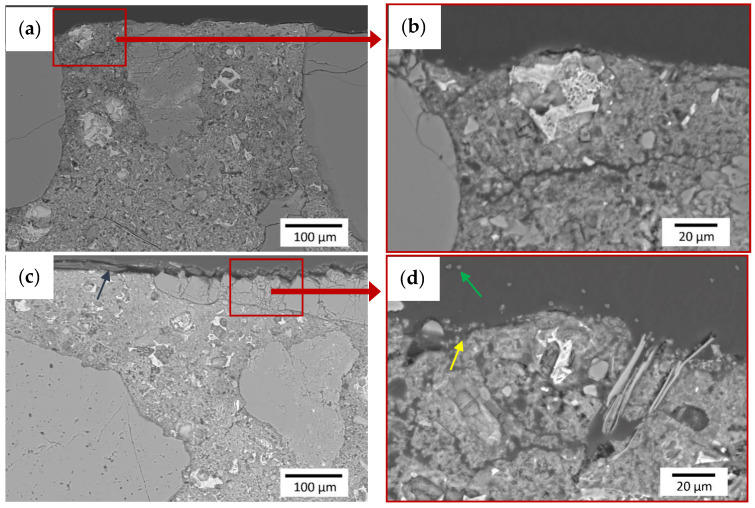
Microstructure of the interphase between the epoxy resin and substrate: (**a**,**b**) unmodified sample, (**c**,**d**) sample modified with 0.5 wt % of polypropylene fibers. The test was conducted using SEM and the BSE detector (yellow arrow—resin-filled pores on the surface of the sample, green arrow—cross-section of polypropylene fibers).

**Figure 17:**
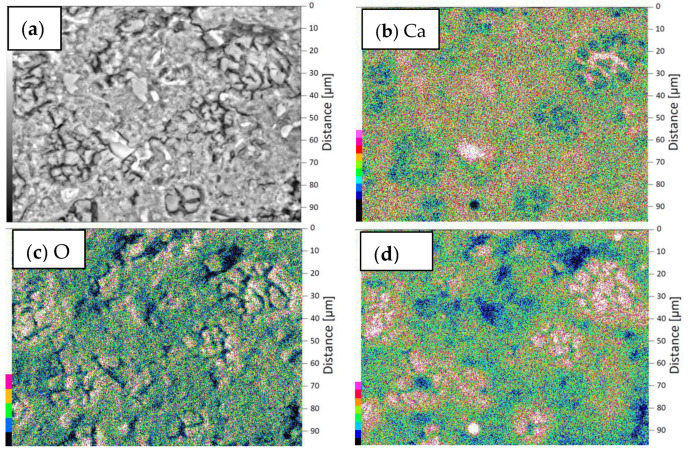
EDX mappings of chemical elements in the subsurface layer of the substrate of the reference sample: (**a**) area of the performed distribution, (**b**) images of Ca incidence, (**c**) images of O incidence, (**d**) images of Si incidence.

**Figure 18:**
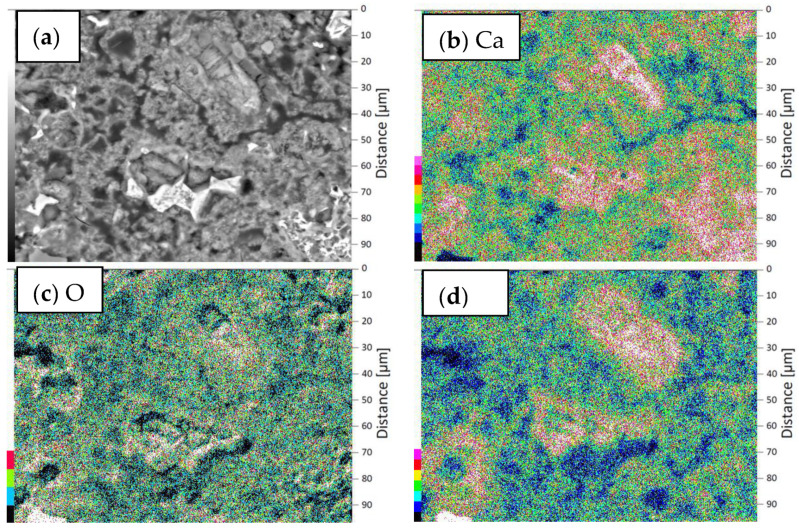
EDX mappings of chemical elements in the subsurface layer of the substrate of the sample modified with 0.5 wt % of polypropylene fibers: (**a**) area of the performed distribution, (**b**) images of Ca incidence, (**c**) images of O incidence, (**d**) images of Si incidence.

SEM investigations were carried out for the reference sample and for the sample with the coating with the addition of 0.5 wt % of polypropylene fibers. Tests were performed for coating samples on the cement mortar. The microstructures of the interphase between the epoxy resin and cement substrate at a magnification of ×200 and ×750 are presented in Figure 16. It was found from Figure 16 that the epoxy resin thoroughly covers the surface of the substrate (Figure 16a,b). Figure 16d shows that the uneven surface of the substrate is well filled by the resin (yellow arrow) and also that the fibers (green arrow) embedded throughout the entire cross-section of the sample are visible. A slight accumulation of fibers at the substrate’s surface was also identified. On this basis, it can be concluded that the addition of polypropylene fibers may have a positive effect on the pull-off strength result, provided that the fibers are carefully separated. From the results obtained using SEM with EDS, maps of the intensity of the occurrence of individual elements at the depth of the substrate were created, starting from the contact surface of the coating with the substrate (Figure 17 and Figure 18).

When comparing the results presented in Figure 17 and Figure 18, it can be seen that the subsurface layer of the substrate of the modified sample has a higher content of calcium and silicon compared to the reference sample (more darker areas). The main components of the aggregate are Si and Ca, so in places with a low content of Ca and Si (pale areas) there are pores that should be filled with epoxy resin. This is important to obtain the appropriate pull-off strength of the coating. It is worth mentioning here that polypropylene fibers consist mainly of carbon, which is impossible to detect using EDS. On the other hand, epoxy resin consists mainly of carbon, hydrogen and oxygen. When comparing the O images in both figures (Figure 17 and Figure 18), a slightly higher oxygen content in the unmodified sample can be seen. However, oxygen is one of the major constituents of the binder in the substrate, so this difference is not necessarily due to the presence of epoxy. Thus, in order to investigate the effect of the addition of polypropylene fibers on the possibility of penetration of the epoxy resin into the substrate, the porosity of the subsurface layer of the substrate should be examined.

**Figure 19:**
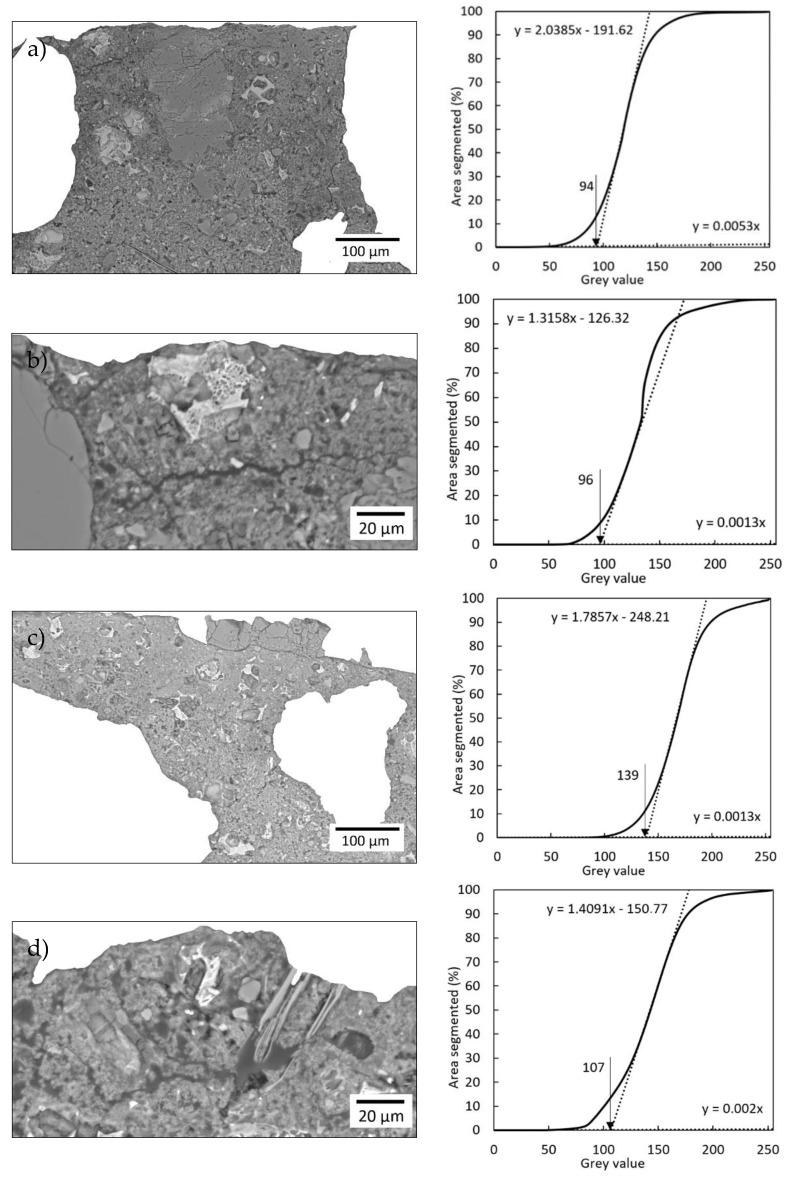
Images at x200 magnification after processing using the ImageJ program and cumulative greyscale histogram for: (**a**,**b**) unmodified sample, (**c**,**d**) sample modified with 0.5% of polypropylene fibers.

**Figure 20:**
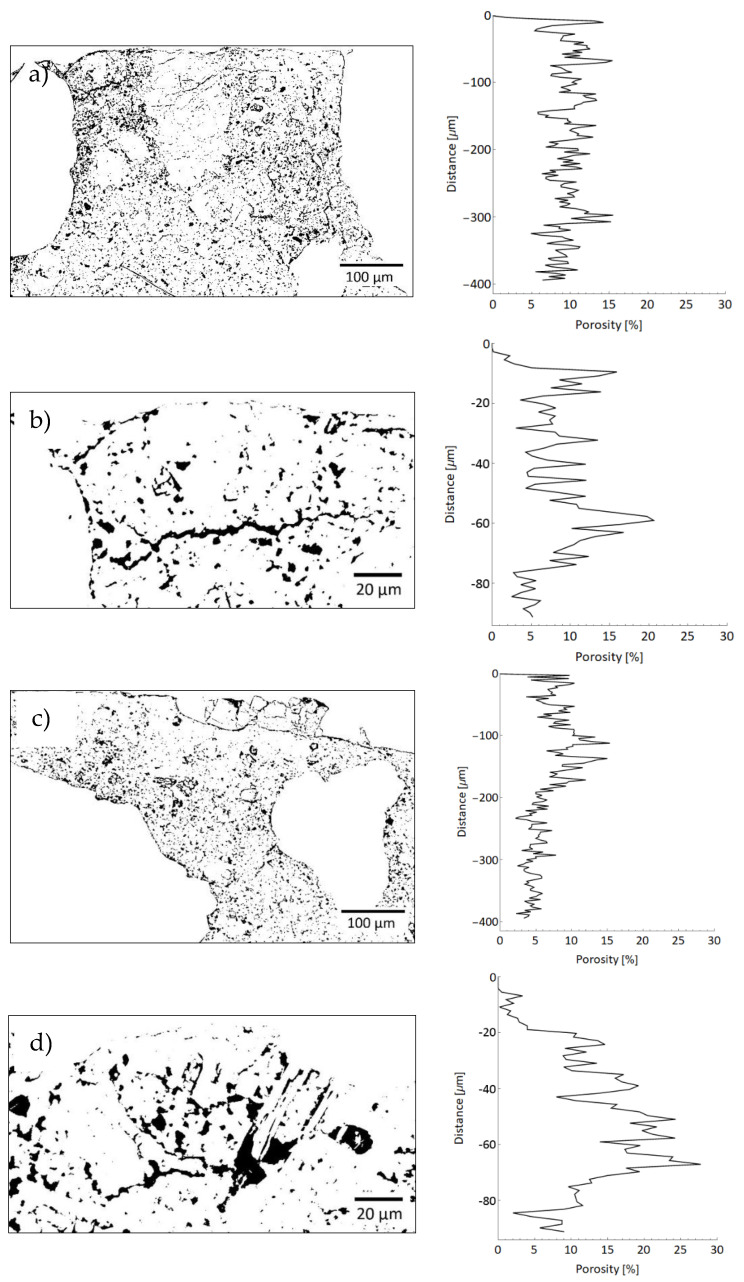
Pores in the tested sample (black pixels) and graphs of the fractional share of pores along the sample’s height for: (**a**,**b**) unmodified sample, (**c**,**d**) sample modified with 0.5% of polypropylene fibers.

Figure 19 shows the cross-sections of the sample’s height at ×200 and ×750 magnification after being processed using the ImageJ program according to the procedure described in [37]. The coating and aggregate were removed from the images, and the optimal “threshold” value of the pores was then determined on the cumulative grayscale histogram (Figure 19). The value of the “threshold” should be determined separately for each image. The “threshold” value means that all the pixels in colors on the gray scale from 0 to the “threshold value” are considered as pores, and the black color is assigned to them using the ImageJ program (Figure 20). Finally, the porosity plots were constructed from the obtained images using the Wolfram Mathematica program (Figure 20).

When comparing the graphs of porosity along the height at ×200 magnification of the surface layer of the substrate (Figure 19a,c), it can be seen that the sample modified with 0.5% of polypropylene fibers has a similar content of pores in comparison to the unmodified sample (values oscillate between 5% and 15%). On the other hand, when comparing the porosity diagrams for the subsurface layer of the substrate at ×750 magnification, a higher content of pores in the modified sample can be seen in comparison to the unmodified sample (by about 5 percentage points). On this basis, it can be concluded that the addition of 0.5% of polypropylene fibers generally does not hinder the penetration of the epoxy resin into the substrate. This is evidenced by the similar percentage of pores down to a depth of about 220 μm (Figure 20a,c). However, very small areas may appear, where the pore content will increase, as evidenced by the higher percentage of pores to a depth of about 70 μm in the modified sample (Figure 20d).

## 4. Short Discussion and Final Conclusions

The combination of polypropylene fibers with epoxy resin is the subject of many articles. However, there is little work directly related to construction, more specifically epoxy coatings. In the present article, the influence of high temperature on such coatings was investigated, the mechanical properties of composites containing resin and polypropylene fibers were examined and the impact of drilling on such coatings was observed. The research results presented in this article differ mainly in the purpose—the assumption was to obtain the highest possible pull-off strength result, which was achieved with a low amount of additive: 0.5–1%. In the above-mentioned materials, the optimal addition of fibers was from 10% to even 40% (composites). All works had a similar thickness of the coating: 3–5 mm. In general, the topics of all works were subordinated to a specific goal, in this case it was also achieved. Literature research and these research give promising evidence that polypropylene fibers can also improve some of its parameters of epoxy resins used in industrial flooring.

Considering the above, this article describes the effect of adding polypropylene fibers to primer on the pull-off strength of epoxy resin coatings. Tests were carried out on substrates made of cement mortar and cement slurry. The primer used to make the coating was made of epoxy resin modified with the addition of 0.5%, 1%, 1.5% and 2% of polypropylene fibers. The research results were compared with the reference sample (without the addition of fibers). The main conclusions of the study are:

- the optimum content of polypropylene fibers was found to be equal to 0.5–1.0 wt % of the mass of the resin. It was found that 1 wt % of fibers was optimum for epoxy resin laid on the cement slurry, while 0.5 wt % of fibers was optimum for epoxy resin laid on the cement mortar. The addition of a higher content of polypropylene fibers resulted in the reduction of the pull-off strength of coatings when compared to the reference sample,
- the results of the velocity of ultrasounds as a function of the sample’s height confirm that the sample without polypropylene fibers better filled the pores in the resin area (the speed turned out to be higher) than in the case of adding 0.5 wt % of polypropylene fibers,
- the chemical analysis performed using scanning electron microscopy (SEM) and energy-dispersed x-ray spectrometry (EDS) showed that the subsurface layer of the substrate of the sample with 0.5 wt % of polypropylene fibers has a higher content of calcium and silicon when compared to the reference sample,
- the microstructural analysis performed using SEM shows that the epoxy resin modified with polypropylene fibers covers the surface of the substrate very well. This is confirmed by the high values of the pull-off strength obtained during the tests, even in the case when there was a small amount of fibers. However, further analysis also shows that the addition of 0.5 wt % of polypropylene fibers does not hinder the penetration of the epoxy resin into the substrate. This is evidenced by a similar percentage of pores along the sample’s height.

## Figures and Tables

**Table 1:** Measurement results of the diameters of polypropylene fibers ($\bar{x}$ —mean, SD—standard deviation, Q1—first quartile, Q3—third quartile, IQR—interquartile range).

$\bar{x}$	SD [μm]	Median [μm]	Q1 [μm]	Q3 [μm]	IQR [μm]
20.4	1.8	20.3	19.3	21.5	2.2
